# Supplementary material for: Effect of friction on oxidative graphite intercalation and high-quality graphene formation
Source: Nat Commun. 2018 Feb 26;9:836. doi: 10.1038/s41467-018-03211-1 (PMC5826935; doi:10.1038/s41467-018-03211-1)
Supplement: Supplementary file 1 — Supplementary Information [file 41467_2018_3211_MOESM1_ESM.pdf]

Supplementary Information

Effect of friction on oxidative graphite intercalation and high-quality graphene formation

Steffen Seiler[1], Christian E. Halbig[2], Fabian Grote[2], Philipp Rietsch[2], Felix Börrnert[3], Ute Kaiser[3], Bernd Meyer[1]*, and Siegfried Eigler[2]*

1) Interdisciplinary Center for Molecular Materials (ICMM) and Computer-Chemistry-Center (CCC), Friedrich-Alexander-Universität Erlangen-Nürnberg (FAU), Nägelsbachstraße 25, 91052 Erlangen, Germany
Fax: +49 9131 85 26565, E-mail: bernd.meyer@chemie.uni-erlangen.de

2) Department of Chemistry and Pharmacy and Institute of Advanced Materials and Processes (ZMP), Friedrich-Alexander-Universität Erlangen-Nürnberg (FAU), Henkestraße 42, 91054 Erlangen, Germany
and
Institute of Chemistry and Biochemistry, Freie Universität Berlin, Takustraße 3, 14195 Berlin, Germany
Fax: +49 30 838 61851, E-mail: siegfried.eigler@fu-berlin.de

3) Materialwissenschaftliche Elektronenmikroskopie, Universität Ulm, Albert-Einstein-Allee 11, 89081 Ulm, Germany

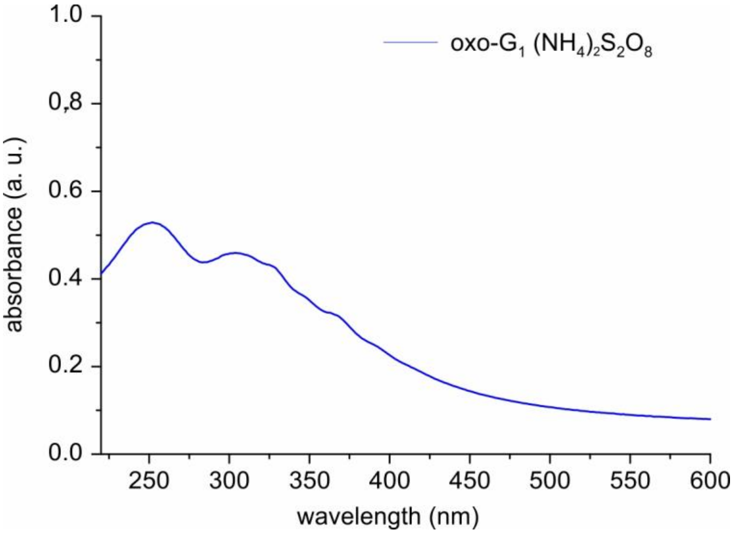

Supplementary Figure 1 | UV-Vis spectrum of oxo-G_1 prepared from NG1 using ammonium persulfate as oxidant. Single layers of oxo-G_1 were enriched by centrifugation technique. Solvent: 1/1 (by volume) of water/methanol.

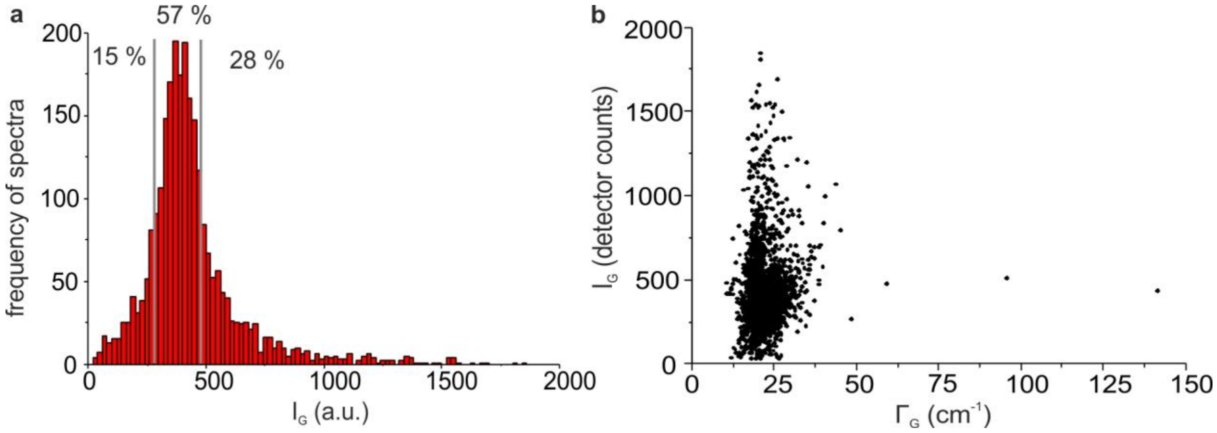

Supplementary Figure 2 | (**a**) Histogram of the frequency of I_G generated from statistical Raman spectroscopic data. The histogram reveals 15% of area is due to substrate and overlap with single layer graphene. About 57% of Raman spectra relate to single layer graphene and 28% of Raman spectra relate to few-layer graphene. (**b**) I_G *vs.* Γ_G: reference measurements reveal that single layer graphene can be related to roughly 300-500 detector counts.

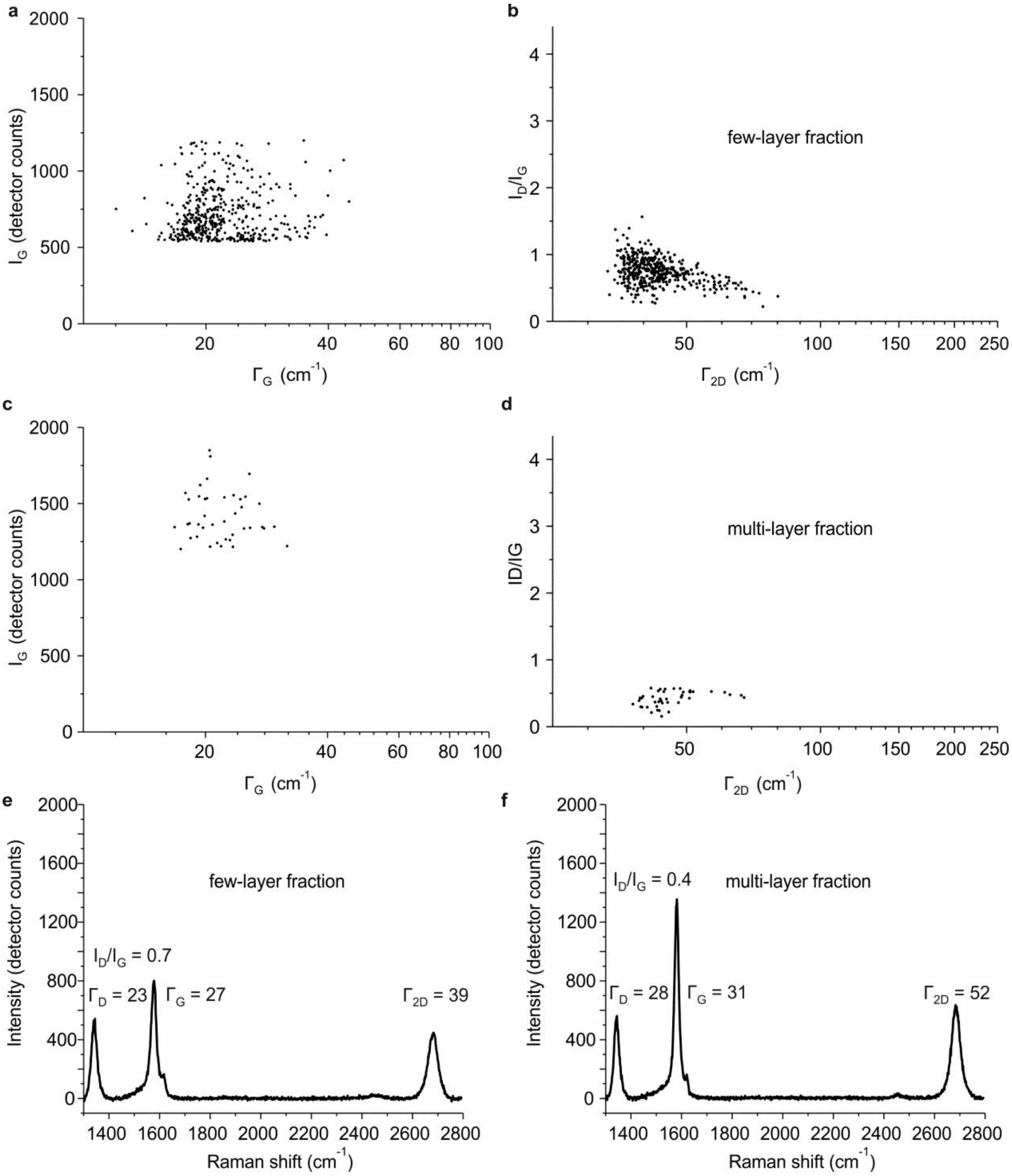

Supplementary Figure 3 | (**a**) I_G *vs.* Γ_G: only I_G values between 540 and 2000 detector counts are included. Those spectra stem mainly from few-layers. (**b**) I_D/I_G *vs.* Γ_{2D}: few-layer fraction, filtered by the I_G value (between 540 and 1200 detector counts; $I_D/I_G = 0.74 \pm 0.2$; $\Gamma_{2D} = 44 \pm 7$ cm^{-1}). (**c**) I_G *vs.* Γ_G: only I_G values higher than 1200 detector counts are included. Those data stem mainly from multi-layers. (**d**) I_D/I_G *vs.* Γ_{2D}: multi-layer fraction, filtered by the I_G value (> 1200 detector counts; $I_D/I_G = 0.42 \pm 0.2$; $\Gamma_{2D} = 47 \pm 7$ cm^{-1}). (**e**) Raman spectrum of few-layer graphene with I_D/I_G ratio of 0.7 and I_G of about 800 detector counts. (**f**) Raman spectrum of multi-layer graphene with I_D/I_G ratio of 0.4 and I_G of about 1400 detector counts.

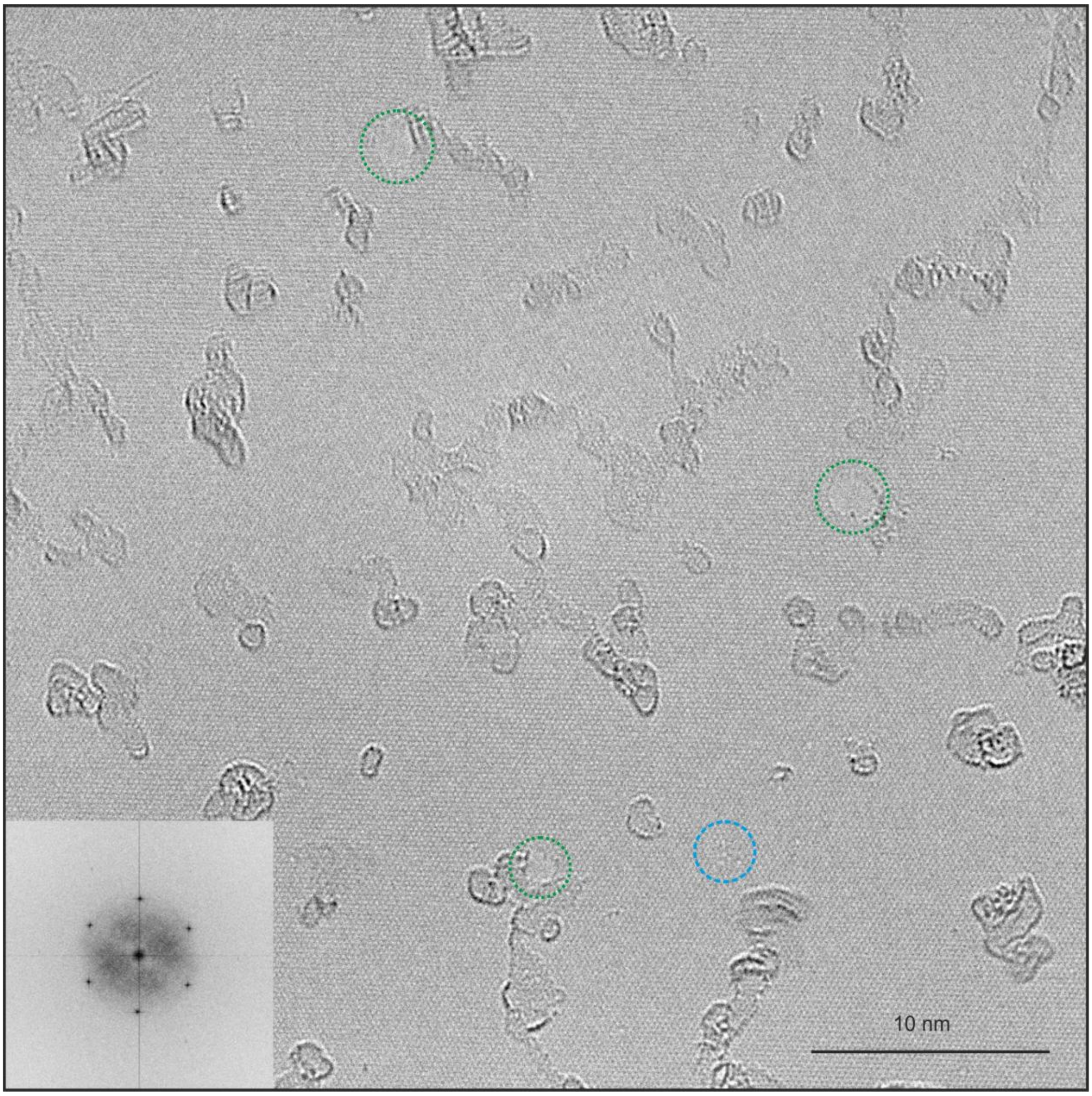

Supplementary Figure 4 | Large-scale TEM image shows the resolved carbon lattice of graphene next to amorphous impurities; lattice defects are mainly point defects.

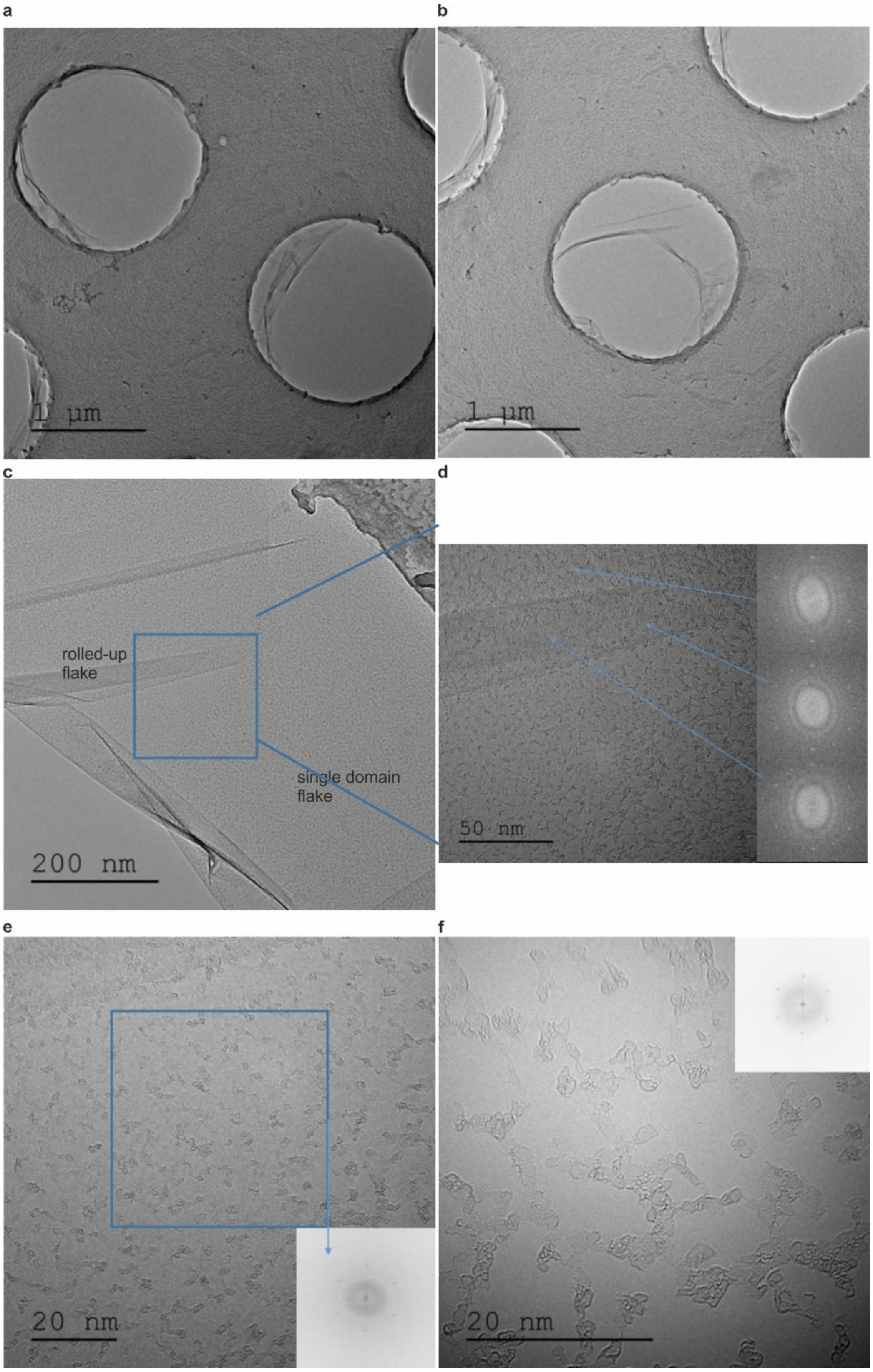

Supplementary Figure 5 | (**a**) and (**b**) Overview images of TEM grids with micrometer-sized holes covered with oxo-G_1; (**c**) enlargement of oxo-G_1 membrane shown in (b) and (**d**) further enlargement with diffractograms revealing a rolled-up slightly rotated second single-layer flake on top of a single-layer oxo-G_1. (**e**) Further magnification of the single-layer region in (d). (**f**) A far-away region of single-layer oxo-G_1.

Supplementary Note 1

Stability of oxo-species

A thermodynamic integration (TI) simulation, also often referred to as 'blue moon ensemble' technique,[1,2] was performed to confirm that oxo-species remain attached to the carbon scaffold at oxidation levels higher than C_{30}^{+}. With this method, free energy differences between an initial and a final reaction configuration can be calculated. To this end, a reaction coordinate ξ, connecting initial and final state by a continuous path, is chosen and divided into a set of discrete sampling points. For each value of ξ a constrained MD run is performed, in which the reaction coordinate ξ is kept fixed at its initial value. The force required to keep the reaction coordinate ξ at its fixed value is monitored and the average is taken (mean force). The free energy difference between the initial and final reaction state is then obtained by numerical quadrature of the mean force on the constraint over the discrete sampling points.

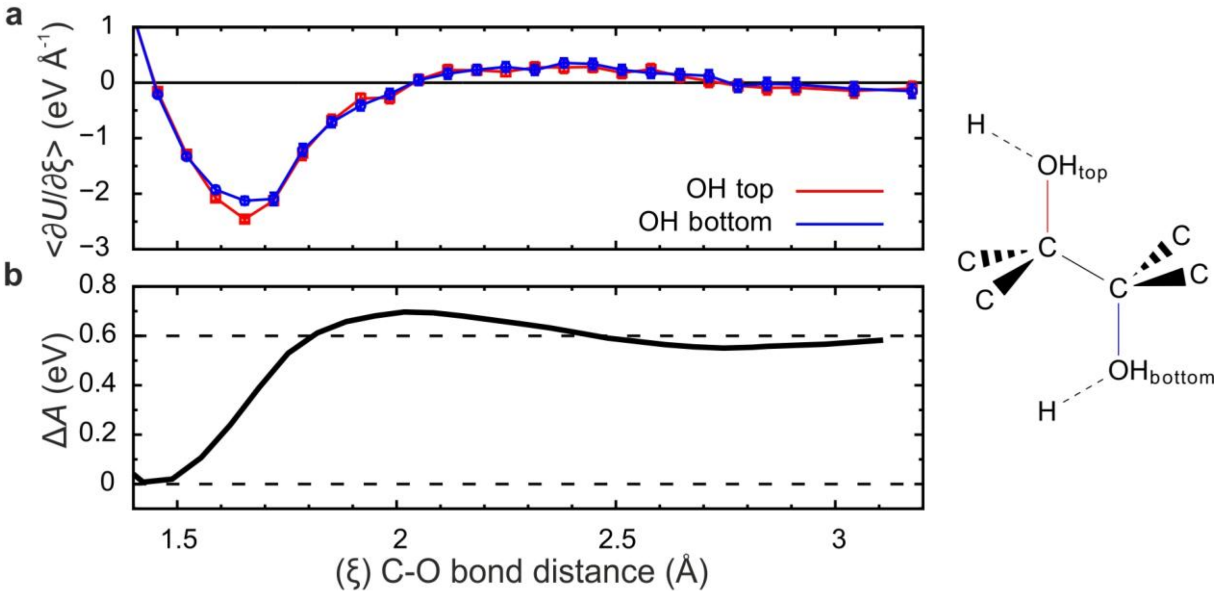

Supplementary Figure 6 | (a) The red and blue curves depict the mean force acting on the reaction coordinate ξ (the C-O bond distance) for the detachment of the upper and lower OH group, respectively. The similarity of the curves confirms the convergence of the simulation procedure. Error bars represent the standard error of the mean, assuming a statistical inefficiency[3,4] of 100, as determined by a block analysis of selected runs. (**b**) Integrated mean force, which gives the free energy profile along the reaction pathway ξ. The average of the mean forces of the two simulations shown in the top panel was used for the integration.

The TI simulation was performed for the orthorhombic ($5 \times 3\sqrt{3}$) supercell with 60 carbon atoms and 4 attached OH groups (see central column of **Fig. 5** in the manuscript). After the spontaneous desorption of two OH groups in the course of the initial stage of the MD simulations, the two water molecules formed in this process were removed from the liquid layer in order to reduce dilution effects on the outcome of the TI simulation. As reaction coordinate ξ we used the C-O distance between the hydroxyl oxygen atom and its covalent carbon neighbor. Subsequently, a third OH group was forcibly removed from the carbon scaffold by a stepwise increase of ξ. The force acting on the constraint was monitored for 3 ps at 25 equidistant sampling points (see top panel in **Supplementary Fig. 6**). The integration of the mean forces yields a free energy difference between the initial state (attached OH group) and the final state (OH group dissolved in the sulfuric acid layer in form of a hydronium ion) of about 0.6 eV (about 60 kJ mol^{-1}), thus confirming the stability of the remaining OH groups after the oxidation state of C_{30}^{+} has been reached.

Supplementary Note 2

Electronic structure

In order to understand in more detail why one positive charge per about 30 carbon atoms is the preferred oxidation state of stage-1 GIC we performed an analysis of the electronic structure for the non-oxidized GIC-AA and the oxidized ox-GIC(C_{30}^{+})-AA system. To this aim we calculated projected density of states (DOS) from representative snapshots of our MD simulations with the small $5 \times 3\sqrt{3}$ supercell containing 60 carbon atoms. 50 snapshots at intervals of 2 ps were taken from the 100 ps trajectories. For each configuration a SCF calculation with a (8,8,1) Monkhorst-Pack *k*-point mesh was performed and the wave functions were projected onto a minimal atomic basis. A Gaussian smearing with a smearing width of 0.01 Ry was used for the DOS calculation. The DOS plots averaged over the 50 snapshots are shown in **Supplementary Fig. 7a** and **b**. In addition, iso-electron-density contour surfaces of states integrated up to approximately 2 and 4 electrons below the respective Fermi levels are included.

The DOS plots show that oxidizing the stage-1 GIC to C_{30}^{+} leads to a downward shift of the Fermi level of about 1 eV. The states, which are depopulated by removing 2 electrons from a

unit cell with 60 carbon atoms, are the π-states of the carbon scaffold (see contour plots in **Supplementary Fig. 7c** and **e**). For the oxidized GIC-C_{30}^{+}, however, oxygen states of the sulfuric acid molecules are already close below the Fermi energy. Thus, going to higher oxidation states than C_{30}^{+} by removing additional electrons would not only depopulate carbon states but also oxygen states of the sulfuric acid molecules, *i.e.*, it would lead to a partial oxidation of the sulfuric acid (see **Supplementary Fig. 7d** and **f**). This highly unfavorable situation indicates that at oxidation states beyond C_{30}^{+} it now becomes more favorable for oxo-species to remain attached on the graphite scaffold.

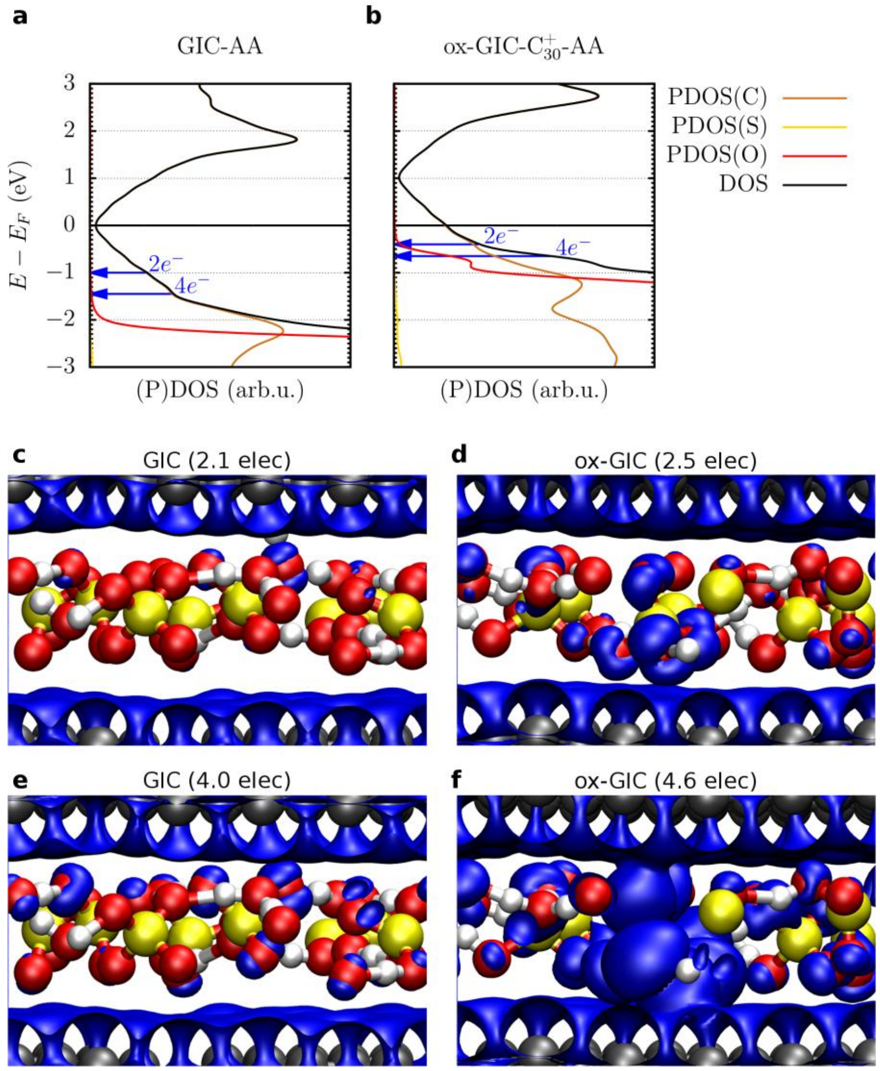

Supplementary Figure 7 | (a) and **(b)** show the projected density of states for GIC-AA and ox-GIC(C_{30}^{+})-AA. The horizontal black solid line at zero indicates the Fermi level. Electronic states between the Fermi level and the blue arrows were added up for obtaining the iso-electron-density contour surfaces (density of 10^{-4} a.u.) shown in the bottom panels (**c-f**).

Supplementary Note 3

Convergence of the friction coefficient

The dependence of the Green-Kubo force auto-correlation function $\lambda_{GK}(t)$ on the overall length of the MD simulation was tested by the following procedure: from the full MD trajectory of 70 ps only the last 10 ps, 20 ps, etc., were used for the evaluation of $\lambda_{GK}(t)$. The corresponding graphs for the large GIC-AA supercell with lateral size of $10 \times 6\sqrt{3}$ graphite lattice constants (*i.e.*, 240 carbon atoms) are shown in **Supplementary Fig. 8**. A steady convergence of the graphs with increasing length of the evaluated segment of the trajectory can be seen. Convergence is achieved after a MD simulation time of about 40 ps, indicating that our 70 ps simulations are long enough for a reliable determination of the friction coefficient λ.

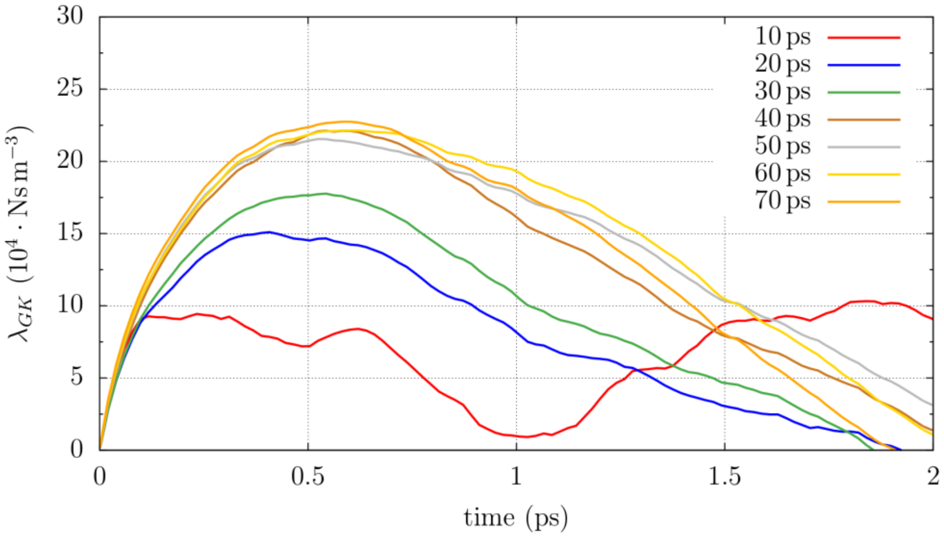

Supplementary Figure 8 | Convergence of the Green-Kubo force auto-correlation function $\lambda_{GK}(t)$ with simulation time for the GIC-AA supercell. The different graphs were calculated from the final part of the MD trajectory with length indicated in the inset.

To assess the statistical error in the Green-Kubo auto-correlation function $\lambda_{GK}(t)$ for our simulations (error bars in **Fig. 6e** of the manuscript), we calculated the variance σ^2 in the mean using block averaging. Starting points for the block averaging were taken according to the statistical inefficiency parameter *s* as introduced by Friedberg and Cameron.[3,4] The statistical inefficiency describes after how many time steps an MD trajectory provides new, uncorrelated information for a property $A(t)$. In our case, the property of interest is the auto-correlation function of the carbon force components parallel to the graphite sheets:

$$A(t) = \int_0^t \langle \mathbf{F_p}(t') \cdot \mathbf{F_p}(0) \rangle \, dt' \quad .$$

To determine the statistical inefficiency s, the MD trajectory of length τ_{run} is broken up into n_{b} blocks, each of length τ_{b}. The variance in the block means

$$\sigma^2(\langle A \rangle_{\mathrm{b}}) = \frac{1}{n_{\mathrm{b}}} \sum_{b=1}^{n_{\mathrm{b}}} (\langle A \rangle_{\mathrm{b}} - \langle A \rangle_{\mathrm{run}})^2$$

is expected to be inversely proportional to τ_{b} at large τ_{b}, as the blocks become large enough to be statistically uncorrelated.[4] The ratio between $\tau_{\mathrm{b}} \sigma^2(\langle A \rangle_{\mathrm{b}})$ in the limit of large block size τ_{b} and the variance $\sigma^2(A)$ expected on the assumption of uncorrelated Gaussian statistics is the statistical inefficiency s of $A(t)$:[3,4]

$$s = \lim_{\tau_{\mathrm{b}} \to \infty} si(\tau_{\mathrm{b}}) = \lim_{\tau_{\mathrm{b}} \to \infty} \frac{\tau_{\mathrm{b}} \sigma^2(\langle A \rangle_{\mathrm{b}})}{\sigma^2(A)} \quad .$$

The function $si(\tau_{\mathrm{b}})$ with different upper limits in the force auto-correlation integral is shown in **Supplementary Fig. 9** for the GIC-AA simulation. The plateau value indicates a statistical inefficiency of about s = 1000 MD time steps.

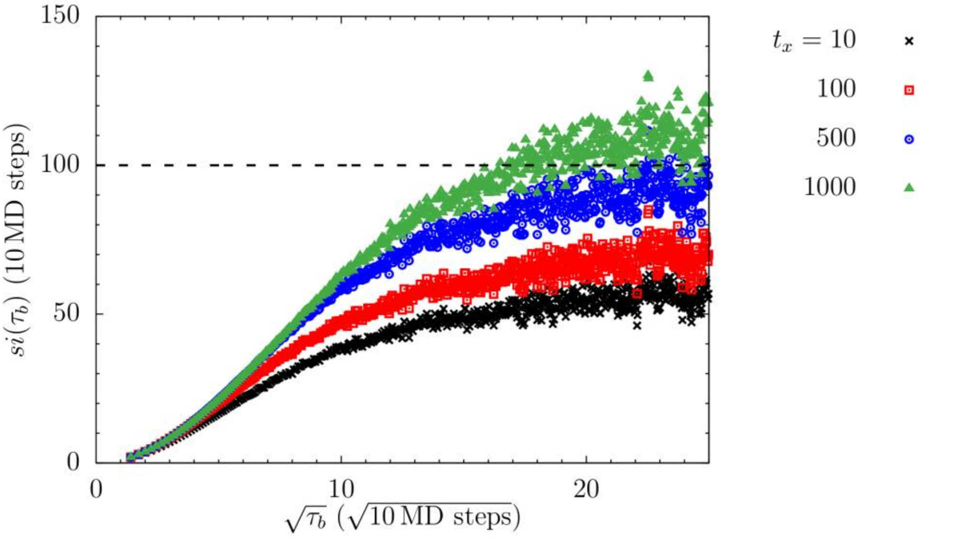

Supplementary Figure 9 Calculation of the statistical inefficiency s for the GIC-AA simulation. The graphs were determined for different integration times t_x (given in multiples of MD steps) of the force auto-correlation function $A(t_x)$. From the plateau region (dashed black line) a value for s of about 1000 MD steps can be derived.

Supplementary Note 4

Influence of oxidation on the orientation of the sulfuric acid molecules

The comparison of the atomic density distributions from the GIC-AA and ox-GIC(C_{30}^{+})-AA simulations in **Supplementary Fig. 10** clearly demonstrates a preferred reorientation of the sulfuric acid molecules in the liquid layer upon oxidation of the graphite sheets. OH groups of the sulfuric acid molecules less frequently stick out of the liquid, which leads to a depletion in the density of the hydrogen atoms close to the carbon layers. The interaction between the positively charged hydrogen atoms of the sulfuric acid OH groups and the electron-rich

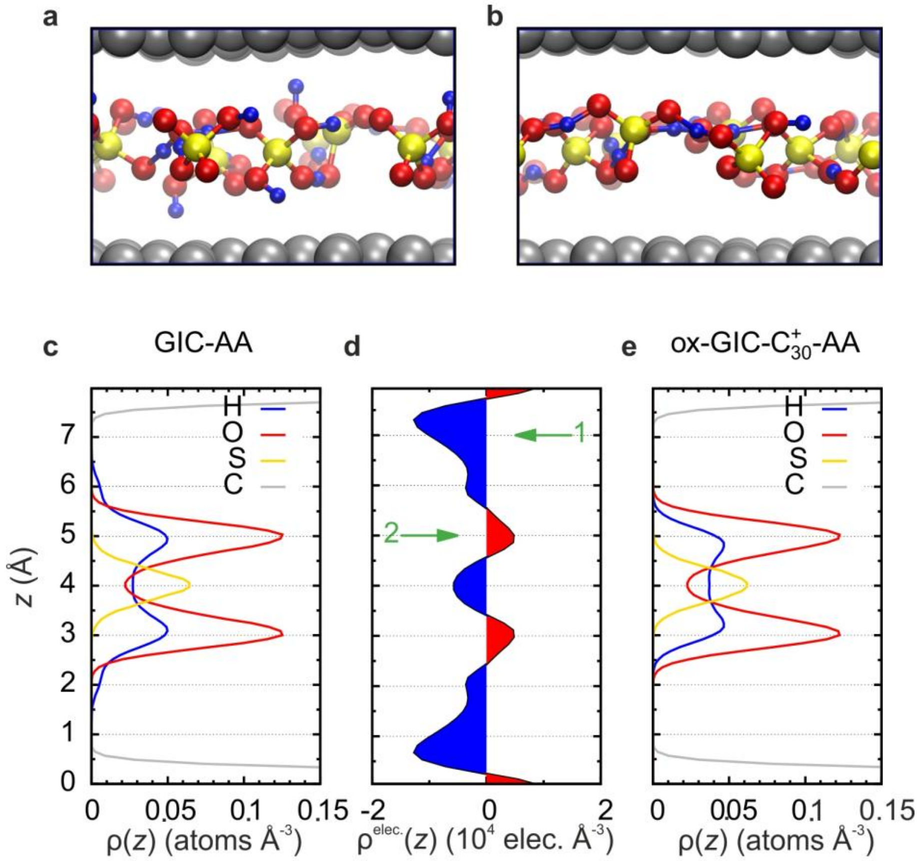

Supplementary Figure 10 | Representative snapshots from (**a**) the GIC-AA and (**b**) the ox-GIC(C_{30}^{+})-AA simulations. The atomic species are color-coded according to the legends in (c,e). (**c,e**) Density of atoms at different heights z above the carbon layer center of mass, obtained from 70 ps MD runs. (**d**) Electron density difference profile $\Delta\rho^{\text{elec.}} = \rho^{\text{elec.}}$(ox-GIC($C_{30}^{+}$)-AA) - $\rho^{\text{elec.}}$(GIC-AA), computed from averaging the electron density from 50 snapshots along the MD trajectories. All data depicted in panels (c-e) were symmetrized with respect to the midplane.

carbon π-system, which is mainly responsible for the corrugation of the free energy surface of **Fig. 6g** in the manuscript and the friction between the sulfuric acid and the graphite layers, is significantly reduced (see also **Supplementary Fig. 11**). This result is further corroborated by the reduction of the electron density in the region of the carbon π-system (green arrow 1 in **Supplementary Fig. 10d**) after oxidation. Furthermore, the electron density difference profile confirms the electron redistribution from the π-system of the carbon layers to the region of the sulfuric acid oxygen atoms upon oxidation (green arrow 2).

Supplementary Note 5

Hydrogen atom probability distribution map

In contrast to the electronegative oxygen atoms, which avoid regions close to the carbon π-system (see **Fig. 6f** in the manuscript), the lateral distribution of the hydrogen atoms given in **Supplementary Fig. 11** reflects their electropositive nature by showing a strong preference to reside close to the carbon scaffold. The same trend as in the oxygen probability distribution maps is found for hydrogen: upon oxidation and alteration of the stacking sequence the probability maps become more diffuse and the corrugation is reduced.

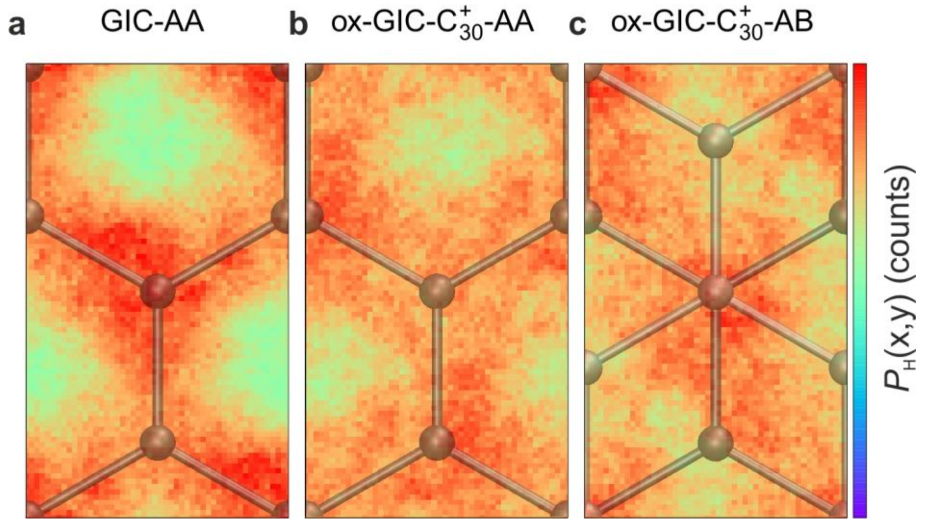

Supplementary Figure 11 | Probability distribution map of hydrogen atoms (same *z*-scale for all subfigures) mapped on a single orthorhombic unit cell for the GIC-AA, ox-GIC(C^+_{30})-AA and ox-GIC(C^+_{30})-AB simulation. Data for these plots were acquired from 70 ps MD runs.

Supplementary Note 6

Influence of stacking on the corrugation of the probability distribution maps

To understand why the corrugation in the probability distribution maps is reduced when changing the stacking of the graphite layers from AA to AB, it is instructive to create two separate maps for the simulation with AB stacking by projecting the atoms from the lower half of the liquid layer onto the bottom graphite sheet and the atoms from the upper half of the liquid layer onto the top graphite sheet. **Supplementary Fig. 12** shows the result for the oxygen probability distribution maps. The separate maps in **Supplementary Figs. 12b** and **c** are basically identical to the probability maps for AA-stacking (see **Supplementary Fig. 12d**). The probability map for AB-stacking (see **Supplementary Fig. 12a**), however, is identical to the average of the maps in **Supplementary Fig. 12b** and **c**. Since here the top and bottom graphite layers are horizontally shifted relative to each other, the resulting probability map will become more diffuse. Thus, any deviation from AA-stacking will reduce the corrugation in the probability distribution maps, and the corrugation will be smallest for maximally displaced graphite sheets, which is AB-stacking.

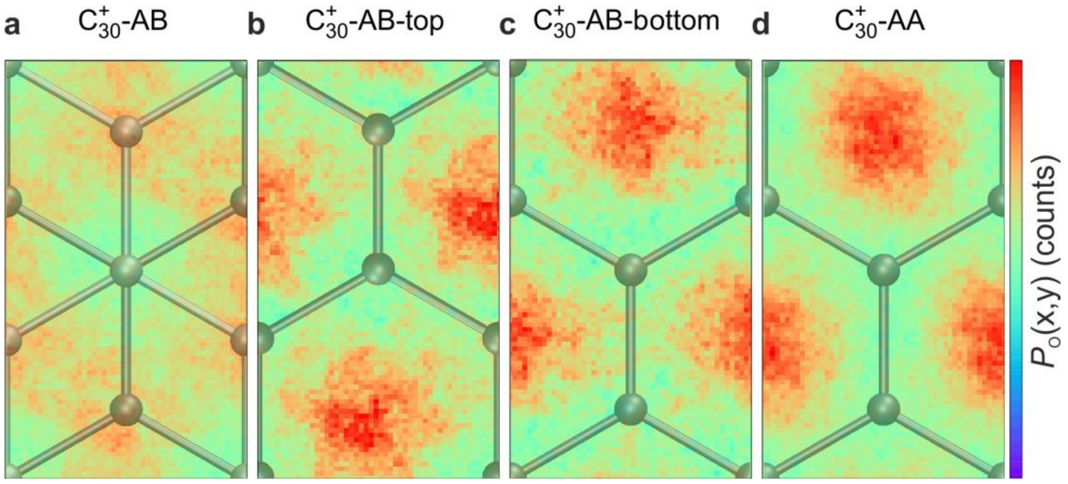

Supplementary Figure 12 | (a-d) Probability distribution of oxygen atoms mapped on a single orthorhombic unit cell for the ox-GIC(C_{30}^{+})-AA and ox-GIC(C_{30}^{+})-AB simulation. **(b,c)** Oxygen atoms in the upper (**b**) and lower (**c**) half of the ox-GIC(C_{30}^{+})-AB supercell have been mapped separately on the top and bottom graphite layer, respectively. To account for the reduced number of sampling points in these evaluation schemes, the counts were multiplied with a factor two. Data for all plots were acquired from 70 ps MD runs. The dividing midplane between the upper and lower half of the sulfuric acid layer was chosen at half the supercell height above carbon atom center of mass.

Supplementary Methods

Raman spectroscopy

The sample was placed on a *x,y* table and it was ensured that the focus of the laser was constant within the scanned area. The laser (532 nm) was focused using an Olympus LMPlanFl 100, NA 0.80 objective lens. The spectrometer was calibrated in frequency using crystalline graphite. For the analysis of oxo-G_1 films, an area of 100×100 μm^2 was scanned using an increment of scanning of 2.5 μm and 0.35 s exposure time. For the characterization of LB films of graphene, an area of 153×153 μm^2 was scanned using an increment of 3 μm and 0.25 s exposure time.

Raman study of process described in Figure 3 of the manuscript

All spectra were recorded after deposition of samples on 300 nm SiO_2/Si wafers. Average spectra of NG1, NG2 and TG from an area of 400 μm^2 were recorded. After GIC preparation average Raman spectra of the same area were measured at laser intensity of about 0.2 mW and variable exposure times meeting a reliable signal/noise ratio. After oxo-G preparation representative maps of samples are recorded. After reduction with HI/TFA Raman maps are recorded to identify Raman spectra of single layer graphene. For NG2, the supernatant was used to identify Raman spectra of graphene.

Supplementary References

(1) Carter E.A., Ciccotti G., Hynes J.T. & Kapral R. Constrained reaction coordinate dynamics for the simulation of rare events. *Chem. Phys. Lett.* **156**, 472 (1989).

(2) Sprik M. & Ciccoti G. Free energy from constrained molecular dynamics. *J. Chem. Phys.* **109**, 7737 (1998).

(3) Friedberg R. & Cameron J.E. Test of the Monte Carlo Method: Fast Simulation of a Small Ising Lattice. *J. Chem. Phys.* **52**, 6049 (1970).

(4) Allen P. & Tildesley D.J. Computer Simulation of Liquids. (Claredon Press, 1987).
